# The Clinical Society of Maryland

**Published:** 1886-08

**Authors:** 


### THE CLINICAL SOCIETY OF MARYLAND.

Officially Reported for THE ATLANTA MEDICAL AND SURGICAL JOURNAL.

STATED MEETING, HELD June 4, 1886.

Dr. Geo. Thomas and Dr. N. T. Carswell were elected to membership in the Society.

Dr. Samuel T. Earle then read

### A REPORT OF TWO AUTOPSIES WHERE THERE WAS INTESTINAL OBSTRUCTION.

He said the two following cases of intestinal obstruction seem to me of sufficient importance to bring before this Society.

*Case 1.*—M. M., colored, insane, female, age 69 years, for many years an inmate of Bay View Asylum. For a week before her death the patient had complained of severe colicky pains in the abdomen, which became very much distended. Death took place rather unexpectedly October 29th, 1884. Autopsy was made October 30th. The body was small, slightly built, emaciated, and the abdomen was very much distended. On opening the abdominal cavity the intestines were found much distended and of a dark-red color. This was particularly the case with the ileum. On endeavoring to lift up the intestines they were found to be fixed in the pelvis, and owing to the great distension it could not be seen what the trouble was. The entire abdominal viscera with the uterus and vagina were removed from the body, and then it was found that the entire ileum from six inches above the valve had passed through a narrow opening that existed in a band of fibrous tissue passing from the left corner of the uterus to the broad ligament. In addition to this constricting band others were found, all being most probably due to an old pelvic peritonitis. The constricted portion of the intestine was very much distended and filled with bloody fluid. Some blood was also found in the peritoneal cavity, and the entire membrane was diffusely stained. A closer examination of the bowel showed that the entire mass twisted once upon its own axis, thus effectually shutting off all circulation in the mesenteric veins. There was no adhesion of the bowel or mesentery to the constricting fibrous band. There was a well-marked prolapse of the vagina and uterus, the cervix uteri lying outside of the vaginal canal. The uterus was five inches long, very narrow, and its canal in places obliterated. The left corner, at which point the constricting band was attached, was very much elevated, and the opposite corner shrunken. In the right broad ligament was a cyst with smooth walls, lined with ciliated epithelium. This cyst was as large as a lemon. This mode of intestinal obstruction, by the bowel passing through an opening made by a band of fibrous tissue, is by no means uncommon.

*Case 2.*—J. D., white, male, age forty-five years. On Thanksgiving day, two days before death, he had eaten very heartily of various substances provided for this feast day. The next day he complained of great pain in the abdomen, and in the evening was suddenly seized with collapse, from which he never rallied. Post-mortem made December 1st, 1884. Body large, strongly built, abdomen distended. Examination of the brain, cord and thoracic viscera revealed nothing of any importance. Both lungs, however, were œdematous. On opening the abdominal cavity there was an escape of gas. Intestines dark and slightly agglutinated. A quantity of pus and yellow floculent matter was found free in the abdomen. In the median line and reaching from the ensiform cartilage to the pubis was a dark distended viscus as large as the arm of a stout man. On further examination this viscus was found to be the cæcum and part of the ascending colon, which had become bent upon itself and turned up so that the caput lay almost touching the stomach, and was completely constricted by the sharp bend it was compelled to make. The small intestine lay beneath it and was greatly distended with gas. The ascending colon was constricted from the same cause. Perforation had taken place in the enormously distended cæcum in three places, and the gut was as thin as paper, and showed evidences of a chronic distension. On opening it a large amount of undigested food was found, raisins, currants, etc., and two large pieces of tendon, one being half as large as the palm of the hand. The man had an abnormally long meso-cæcum, and most probably from distension with gas and undigested food, it had acquired a slight torsion, and as distension continued from the fermentation that was continually going on, the constriction, both of the small intestine and colon, was made more and more complete.

This case represents a form of volvulus that is very rare. There are two in which it may be produced, either by the cæcum being twisted upon itself, or being bent upon itself. In the former instance the cæcum is twisted around on its long or vertical axis, so that its relation to the ascending colon is practically unchanged. In the latter class of cases the cæcum is bent upon itself at right angles to its long axis, which inverts the cæcum, bringing the caput coli in front of the ascending colon and the posterior surface anteriorly. This last variety of cases is represented by the case just reported, except that the caput coli occupied the left hypochondriac region. Distention of the abdomen, generally of an irregular character, was constant, and in every case the cæcum was enormously distended. In the treatment of these cases, as in those of obstruction by bands, laparotomy offers the only rational hope for permanent relief.

Dr. John Chambers thought Dr. Earle had made a valuable point in calling attention to the variation in the length of the mesentery in different persons. He thinks volvulus or hernia is rarely or never seen in persons with short mesenteries; at any rate, he has always failed to produce a hernia in the dead subject where a short mesentery existed, while in the opposite condition of affairs his efforts were successful.

Dr. Randolph Winslow thinks a right colotomy would have been a better suggestion than laparotomy in Dr. Earle's second case.

Dr. Samuel T. Earle, in closing the discussion, said the reason for the gut not collapsing when perforation occurred was that it took place at a point where the gut was pressing closely against the abdominal wall, and this interfered, of course, with any escape of the contained gas.

### CANCER OF THE STOMACH.

Dr. John Chambers showed a specimen of cancer of the stomach from a man æt. 65 years. For about a year he had suffered only from the ordinary symptoms, but lately he was troubled with most intense nausea and vomiting. Four months ago Dr. Coskey diagnosed cancer of the stomach, and his opinion was verified at autopsy.

There were metastices in the liver, but no disease immediately about its aorta. There was no dropsy. The peritoneal cavity, especially its pelvic portion, was studded with carcinomatous nodules. The growth involved the central area of the stomach slightly nearer the pyloric than the cardiac extremity. It gave to the organ an hour-glass shape.

He also showed the head and about four inches of the shaft of the femur that he had resected from a boy æt. nine years. The patient had suffered from a suppurating arthritis for two years.

The portion of the shaft presented was removed without difficulty, as it lay loose in the periosteum.

### OBSTRUCTION OF THE TRANSVERSE COLON.

Dr. R. G. Hall asks the opinion of the Society upon the following case:

A man in whose left hypochondriac region there is a smooth, resonant tumor, not painful upon pressure. The man has no fever, and complains only of a burning sensation at the seat of the enlargement. The burning is relieved by vomiting. He is constipated and has a passage from his bowels only when purgatives are employed, One week since he became very much collapsed, but rallied, and is now much improved.

Dr. J. Edwin Michael said under the circumstances any opinion that might be advanced could be nothing more than a guess. But he would venture as a guess that there is an obstruction in the transverse colon on left side, and that the swelling is the result of the bowel distention and brought about by this obstruction.

Dr. Samuel T. Earle said the evacuations from the bowels in these cases should always be examined, as they often shed much light upon the existing condition. He referred to a case of unsuspected cancer of the rectum that he had recently diagnosed by the contents of the gut, the seat of disease being too far up to be reached by the finger.

Dr. J. H. Branham referred to a case of fæcal impaction that had been mistaken for constriction.

### NÆVUS OF THE TONGUE.

Dr. J. Edwin Michael was recently called upon to remove a nævus from the anterior extremity of the tongue of a young lady. He employed the plan of cutting off the blood supply by a double silver wire ligature placed at its proximal side. On the second or third day it looked as if it were about to slough, but on the sixth day there appeared a rozy zone of healthy looking tongue tissue around the sloughy-looking nævus; closer examination revealed the fact that the wire had cut through the tissue of the tongue at its outer edge, and the portions thus incised had united and circulation was established. From present appearances the nævus will slough out, leaving a ring of healthy tongue tissue.

Dr. G. H. Rohé suggests electrolysis in these cases.

LAPAROTOMY PERFORMED UNDER COCAINE.

Dr. L. McLane Tiffany said he was recently called upon to do an abdominal section, and it occurred to him to use cocaine as a local anæsthetic. He cut off the circulation from the line proposed for the incision by pressing upon the abdomen a wire pessary covered with rubber and bent in the shape of a long narrow rectangle, which would inclose the line in which he proposed to cut. After the circulation was arrested he injected along the line about ℥ss of a four per cent. solution of cocaine. The incision when made was painless until it was extended beyond the line of injection of the salt, when the patient complained of pain.

His second case, in which he employed it, was in an amputation of the penis for epithelioma of the glans. The circulation was arrested by constricting the organ at its proximal end. A four per cent. solution was then injected and the operation completed without pain, exeept when one vessel was being tied, when some discomfort was complained of.

Dr. J. Edwin Michael has used cocaine with most happy results in the smaller surgical operations, and especially in the lesser genito-urinary operations. One of its most effectual fields for action is in the arrest of hemorrhage from mucous surfaces, and especially when occurring in the nasal cavity. He related several cases of extreme hemorrhage from the nose that had been instantly checked by the application of pledgets of cotton soaked in a four per cent. solution of the drug.

The Society then adjourned to meet the first Friday evening of October, 1886.

